# Molecular Mechanisms of Endocrine Resistance in Estrogen-Receptor-Positive Breast Cancer

**DOI:** 10.3389/fendo.2021.599586

**Published:** 2021-03-25

**Authors:** Esmael Besufikad Belachew, Dareskedar Tsehay Sewasew

**Affiliations:** ^1^ Biology, Mizan Tepi University, Addis Ababa, Ethiopia; ^2^ Microbial, Cellular and Molecular Biology Department, Addis Ababa University, Addis Ababa, Ethiopia; ^3^ Immunology, Armauer Hansen Research Institute (AHRI), Addis Ababa, Ethiopia

**Keywords:** acquired resistance, breast cancer, endocrine resistance, endocrine therapy, estrogen receptor, *de novo* resistance

## Abstract

The estrogen receptor is a vital receptor for therapeutic targets in estrogen receptor-positive breast cancer. The main strategy for the treatment of estrogen receptor-positive breast cancers is blocking the estrogen action on estrogen receptors by endocrine therapy but this can be restricted *via* endocrine resistance. Endocrine resistance occurs due to both *de novo* and acquired resistance. This review focuses on the mechanisms of the ligand-dependent and ligand-independent pathways and other coregulators, which are responsible for endocrine resistance. It concludes that combinatorial drugs that target different signaling pathways and coregulatory proteins together with endocrine therapy could be a novel therapeutic modality to stop endocrine resistance.

## Introduction

The estrogen hormone is important in maintaining the function of the reproductive system, bone metabolism, cardiovascular maintenance, central nervous systems, and lubrication of the vaginal lining. Overexpression of the estrogen hormone is associated with an increased risk for breast cancer (1, 2).

The function of estrogen in estrogen receptor (ER) positive breast cancer is primarily mediated by ER. The estrogen receptor is a member of the nuclear receptor superfamily and is involved in various developmental and physiological processes (1). Two subdivisions of ER, estrogen receptor α (ERα) and estrogen receptor β (ERβ) are identified (3, 4). Estrogen receptor α (ERα) is predominantly expressed in the uterus and pituitary gland with highest levels in the liver, hypothalamus, bone, mammary gland, cervix, testis, kidney, heart, skeletal muscle, and vagina. Estrogen receptor β (Erβ) expression is high in the prostate and ovary and found exclusively in the granulosa cells (4, 5). Estrogen receptor α (ERα) activation promotes tumorigenesis in different types of cancer, including breast cancer and the role of ERβ is still unclear (6). Therefore, inhibition of the ERα has become one of the main strategies for the prevention and treatment of breast cancer (7). The main strategy for the treatment of ER-positive breast cancer is blocking the action of estrogen by endocrine therapy but limited by the development of resistance (2). Therefore, this review is mainly focused on the principal mechanisms involved in endocrine therapy resistance among ER-positive breast cancer.

## Structure of the Estrogen Receptor

**Figure 1:**
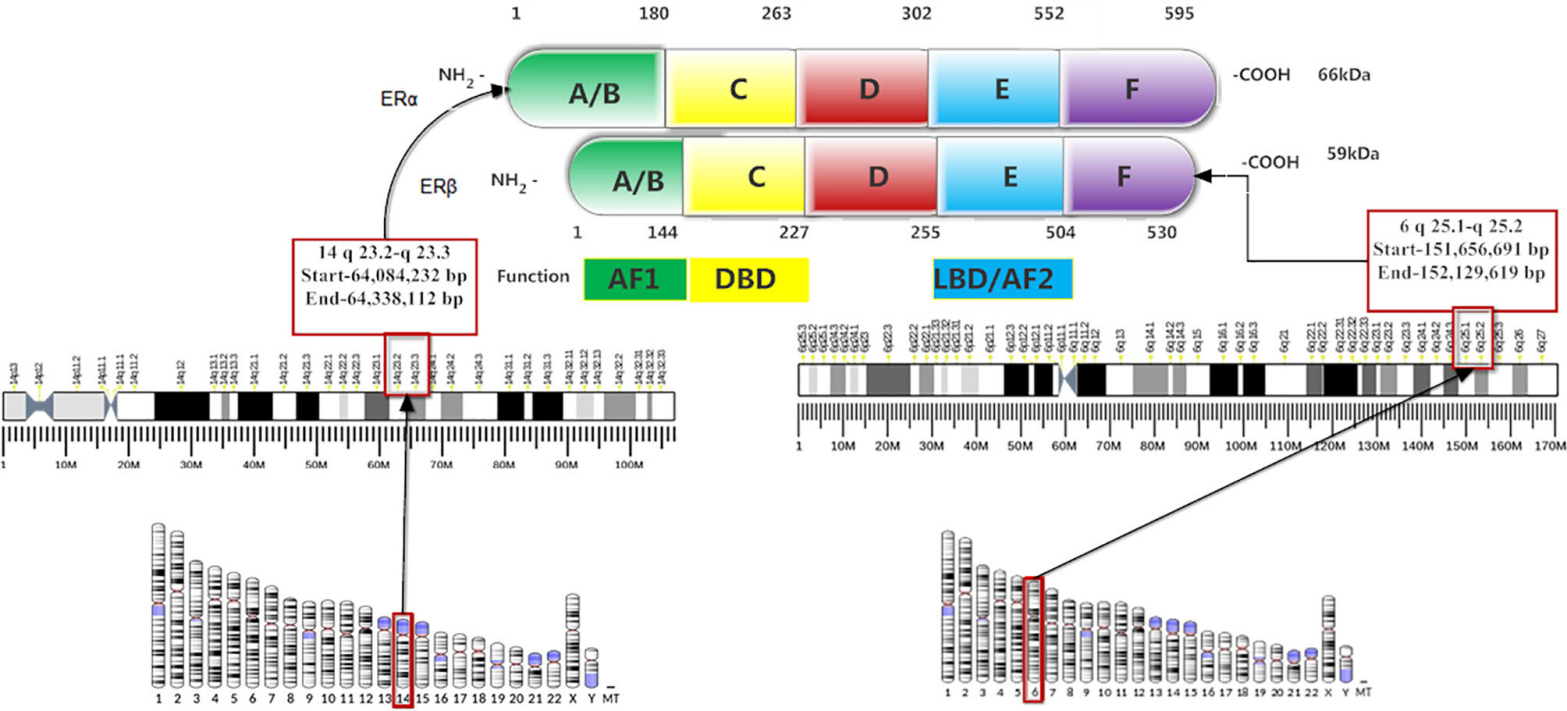
Structure and functional domains of the estrogen receptors.

The structure of ERα and ERβ are similar to other nuclear receptor families, which have four structural and functional domains. These are amino-terminal (A/B domain), DNA binding domain (DBD; C-domain), hinge region (D-domain), and ligand-binding domain (LBD; E-domain) (**Figure 1**). The ERs have an additional fifth domain: the carboxyl-terminal domain (F-domain) whose function is still unclear. DNA binding domain (DBD) and LBD carry 96 and 60% of homology between ERα and ERβ, however, the amino-terminal, hinge region and carboxyl-terminal domains are divergent (4, 5, 8). The amino-terminal (A/B domain) encodes a hormone-independent transcriptional activation function 1 (AF1) (2), and acts synergistically with transcriptional activation function 2 (AF-2) to attain maximum transcriptional activity. The DBD is responsible for ER binding to estrogen response elements (EREs), and the D domain leads to nuclear transport. The LBD contains a dimerization surface and encodes ligand-dependent transcription activation through AF-2 (1, 2, 4, 7).

The genes coding for ERα and ERβ are located on chromosome 14, locus 14q23.2, and chromosome 6, locus 6q25.1, respectively (**Figure 1**). The ERβ has 530 amino acids and 59 kDa molecular weight, while ERα has 595 amino acids and 66 kDa molecular weight (9). Five different isoforms of ERα, such as 62kDa, 53kDa, 46kDa, 45kDa, and 36kDa (10), and five ERβ variants (ERβ1-ERβ5) are detected in breast cancer (11). Both ERα and ERβ1 require ligand binding for ER target gene transcription (12). ERβ1 has gene transcriptional inhibition when signaling through the activator protein 1(AP-1) pathway and its binding with tamoxifen also promotes gene transcription (13). A low level of ERβ1 is an independent marker than ERα level to predict tamoxifen resistance (14).

## Mechanism of Estrogen Receptor Action

The two main mechanisms of ER-dependent gene transcription are estrogen/ligand-dependent and estrogen/ligand-independent (2, 7, 15).

### Ligand-Dependent

In ligand-dependent signaling mechanisms, the binding of estrogen with ER causes a conformational change, which allows various coregulators to stimulate transcription of ER-target genes. The ligand/estrogen-dependent mechanism is further classified into direct genomic or classical, indirect genomic or non-classical, and non-genomic mechanisms of activation (16–19).

#### Direct Genomic/Classical

**Figure 2:**
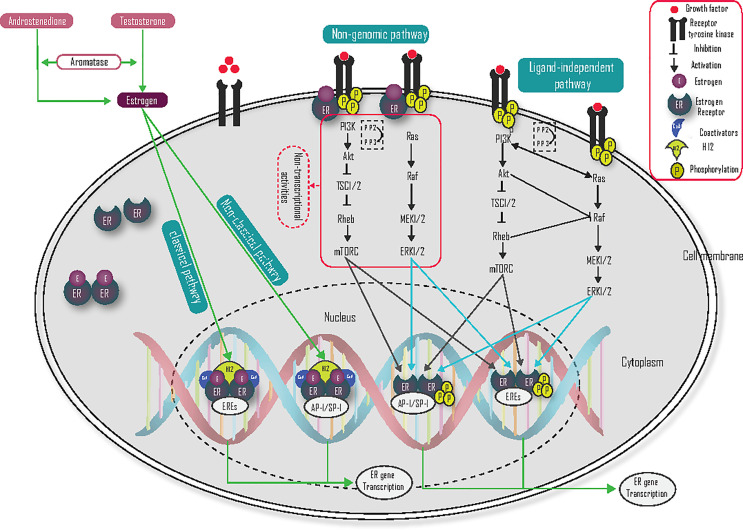
Ligand-dependent and ligand-independent mechanism of action of the estrogen receptor.

The direct genomic or classical pathway regulates the expression of ER target genes by the direct binding of estrogen-activated ERs to DNA binding at EREs (**Figure 2**). During estrogen binding with ER, and the heat shock proteins (HSP70 and HSP90) dissociate ER from this binding in the cytosol, and change their conformation, then migrate as dimers into the nucleus to bind with EREs. This conformational change also allows helix 12 (H12) to accept coactivators and activate gene transcription (10, 20–24).

#### Indirect Genomic/Non-Classical

In indirect genomic/non-classical pathways, estrogen receptors regulate the transcription of genes that do not contain EREs through indirect binding to DNA. The indirect ER binding is mediated by different co-factors (like SP-1, AP-1, and NF-κB) that stimulate gene transcription through interaction with DNA (**Figure 2**) (20, 24). Specificity protein 1 (Sp-1) is the main transcriptional factor that binds with ER and contributes to coactivator recruitment (25). Several genes like low-density lipoprotein receptor, progesterone receptor B, endothelial nitric oxide synthase, GATA binding protein 1, signal transducer and activator of transcription 5, activating transcription factor-2, c-jun, c Fos, ATF-1/cAMP response element-binding protein, nuclear transcription factor-Y, cyclin D1 and the retinoic acid receptor-1α are induced by estrogen *via* the Sp-1 mechanism (10, 25). Activator protein 1 (AP-1) is also another main transcription co-factor that binds with ER and regulates target gene transcription. Genes like insulin-like growth factor-1 (IGF1), collagenase, IGF1-receptor, ovalbumin, and cyclin D1 are induced by the ER-AP-1 binding activation pathway (10), but ERβ inhibits the AP-1 dependent transcription of cyclin D1 (23).

#### Non-Genomic/Membrane-Initiated

The non-genomic ER pathway can occur very quickly and initially independent of genomic gene transcription. This rapid mechanism of action is mediated by the membrane-associated ER. Plasma membrane localization of ER is mediated by heat shock protein 27 (HSP27) (26, 27), and associates with the membrane at caveolae lipid rafts through interactions with caveolin-1, Src, and striatin. The binding of membrane-localized ER and estrogen interact directly with RTK, the p85 regulatory subunit of PI3K, Src, and Shc to activate RAS/RAF/MEK1/2 and ERK1/2, PI3K/Akt/mTOR) signaling pathway (**Figure 2**). These kinase pathways not only induce cell survival and cell proliferation but also phosphorylate ER and its coregulators, which result in the activation of nuclear genomic transcription. Estrogen activates growth factor signaling *via* non-genomic actions of ER and the growth factor signaling, in turn, activates ER, hence forming a vicious cycle (28). Coregulatory proteins such as proline-glutamic acid, leucine-rich protein 1, and metastasis-associated proteins are important to activate non-genomic activity (20, 26, 29, 30). G protein-coupled receptor 30 (GPR30) is also a membrane-localized receptor that has been observed to respond to estrogen to activate rapid signalings (27), such as PI3K and calcium signaling (23). ER-mediated, non-genomic signaling can also regulate nitric oxide, PKC, and calcium flux to promote autophagy, proliferation, apoptosis, survival, and differentiation. The calcium flux *via* membrane-localized ER leads to the activation of kinase pathways (27). As a result, targeting this pathway could be one of the possible treatment strategies to reduce endocrine resistance.

### Ligand-Independent Activation of ER

Growth factors interact with activated receptor tyrosine kinases (RTK) like human epidermal growth factor receptors, insulin-like growth factor-1 receptor (IGF-1R), and the fibroblast growth factor receptor (FGFR), which leads to activation of the phosphatidylinositol 3 kinases (PI3K) signaling pathway (21, 25, 27). Phosphatidylinositol 3 kinase contains a catalytic domain (p110) and a regulatory domain (p85), and it phosphorylates phosphatidylinositol diphosphate (PIP2) to phosphatidylinositol triphosphate (PIP3), which in turn facilitates the phosphorylation of the Akt. Then, Akt activates mTOR *via* the inhibition of tuberous sclerosis 1/2 (TSC1/2). Tuberous sclerosis 1/2 is a tumor suppressor and heterodimer of tuberin and hamartin, which acts as a guanosine triphosphatase activating protein and negatively regulates Rheb-GTP by converting it into its inactive guanosine diphosphate-bound state (31–33). The tumor suppressor gene phosphatase and tensin homolog deleted on chromosome ten (PTEN) have an inhibitory effect on PI3K by dephosphorylating PIP3 to PIP2, and inositol polyphosphate 4-phosphatase type II (INPP4B) is also dephosphorylated PIP3 to PIP26 (31, 32). Activation of the ER-target gene in the PI3K/Akt/mTOR pathway (**Figure 2**) is mediated by phosphorylation of ER on S167 (34). Taken together, activation of the PI3K/Akt/mTOR pathway plays a central role in breast cancer, and blocking of this pathway is an attractive treatment target, especially in endocrine-resistant ER-positive breast cancer.

Growth factors binding with the RTK receptors also lead to activation of the Ras/Raf/MEK/ERK signaling pathway (**Figure 2**) (21, 25, 27). The binding of growth factor with RTK activates RAS. Activated RAS can then bind with RAF and activate the downstream signaling pathway (35). When Raf is activated, its C-terminal catalytic domain can interact with MEK, and its catalytic VIII subregion is phosphorylated at the Ser218 and Ser222 activation loop, which activates MEK1/2. MEK1/2 is further activating ERK1/2 by phosphorylating the Tyr and Thr regulatory sites. Activated ERK1/2 are then translocated to the nucleus and promote phosphorylation of Ser 118 in the AF-1 domain of ER and activate its ER-target gene transcriptional activity (36). This pathway may be also a crucial target for the treatment of endocrine resistance ER-positive breast cancer.

## Endocrine Therapy and Mechanism of Action

Endocrine therapy is the most efficacious drug for the treatment of ER-positive breast cancer patients (37). Based on their chemical structure, endocrine therapy (antiestrogen) is classified into steroidal and non-steroidal antiestrogen, which have no agonist and partial antagonist activity, respectively. The complete loss of estrogenic activity in all tissues by steroidal antiestrogens is not desirable, because estrogen is involved in many other physiological activities, therefore non-steroidal antiestrogen that has partial antagonist activity is preferred (7).

Estrogen receptor-targeted therapy for breast cancer was first used in 1896 by Beatson (3) and currently, at least six distinct therapeutic modalities are established, namely selective ER modulators (SERMs) (tamoxifen, raloxifene, and toremifene), selective ER down-regulators (SERDs), aromatase inhibitors (anastrozole, letrozole, and exemestane), mammalian target of rapamycin inhibitors in combination with aromatase inhibitors, and cyclin-dependent kinases 4 and 6 inhibitors in combination with aromatase inhibitors and cyclin-dependent kinases 4 and 6 inhibitors in combination with SERDs (4). Tamoxifen is the most commonly used SERM treatment and is mostly recommended for premenopausal women (38). It has shown a 31% reduction in the five-year mortality rate among hormone receptor-positive women (39) and around 500,000 women are alive today as a result of tamoxifen therapy alone (40). Aromatase inhibitors are competitive inhibitors of the aromatase enzyme and inhibit the synthesis of estrogen. It is mostly used among postmenopausal patients. Combination of everolimus (mTOR inhibitor) with endocrine therapy is also a breakthrough treatment strategy for previously aromatase inhibitor-treated advanced breast cancer (41–46).

Selective ER down-regulators like fulvestrant have antagonistic effects only and were approved by the FDA in 2007 for the treatment of ER-positive, metastatic breast cancer (46). Fulvestrant is shown to have a binding affinity, which is 100 times greater than tamoxifen (47).

**Figure 3:**
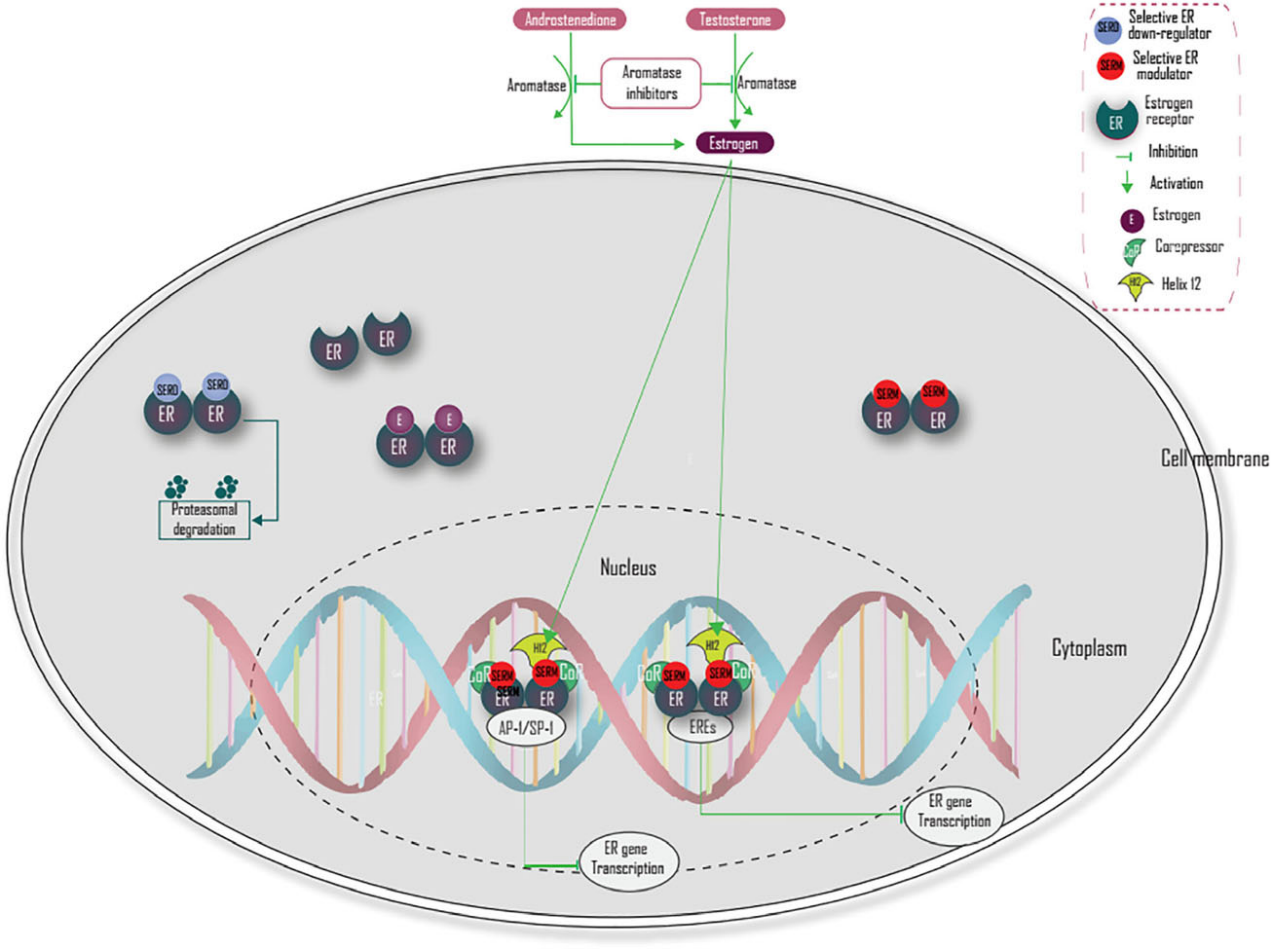
Mechanisms of action of main endocrine treatment.

There are two main strategies to block ER signaling in breast cancer. The first mechanism is through inhibition of estrogen action *via* ER antagonism. The second mechanism is through the reduction of estrogen levels. Selective ER modulators competitively inhibit estrogen action by binding and blocking ER (2, 37). This blocking prevents H12 from capping and causes H12 to occlude the coactivator recognition groove, which prevents coactivator proteins from binding and recruits corepressors that lead to termination of estrogen-activated gene transcription (38). Selective estrogen receptor down-regulators (SERDs) (fulvestrant) compete with estrogen for binding with ER, inhibit ER receptor dimerization, and induces proteasome-dependent degradation of the estrogen receptor (37, 44, 48) (**Figure 3**). However, a combination of endocrine therapy with other signaling pathways like PI3K/AKT/mTOR and Ras/Raf/MEK/ERK and coregulatory factors targeted agent should be the focus of future treatment, especially in endocrine-resistant breast cancer.

## Endocrine Resistance

Endocrine resistance occurs due to both *de novo* and acquired resistance. De novo resistance happens when endocrine resistance develops at the beginning of treatment, while acquired resistance occurs by non-responsive or stimulated growth after endocrine therapy (3).

### *De Novo* or Intrinsic Resistance

Approximately, 30% of ER-positive tumors developed *de novo* resistance to tamoxifen therapy (49). The primary mechanism of *de novo* or intrinsic resistance to endocrine therapy particularly to tamoxifen, is due to lack of expression of ER (4). Recently, a second intrinsic mechanism has been documented in which patients carrying inactive alleles of cytochrome P450/2D6 (CYP2D6) fail to convert tamoxifen to its active metabolite, and are less responsive to tamoxifen (50).

### Acquired Resistance

Acquired resistance occurs after endocrine treatment and the several factors listed below are responsible for this resistance (3).

#### Loss of ER Expression

Approximately 20% of breast cancer patients treated with endocrine therapy lose ER over time (37). Loss of ER expression involves a switch from an initially ER-positive to ER-negative phenotype, as a result, breast cancer is not suppressed by endocrine therapy that specifically targets ER. The transcriptional repression and population remodeling of the ER gene are responsible for the loss of ER expression (3). Transcriptional repression may be due to epigenetic changes, such as aberrant CpG island methylation of the ER promoter and histone deacetylation by histone deacetylase enzyme, resulting in a compact nucleosome structure that limits transcription (51). A small proportion of breast cancers presenting with nonexistent ER gene expression have an intrinsic gain in CpG site methylation (4).

#### Mutation of the ER Gene

Estrogen receptor gene mutations are seen in resistant breast cancer cells (52). The ER mutations are commonly found within the LBD, and the most common hot spot mutation is Tyr537Ser, Tyr537Asn, and Asp538Gly (Y537S, Y537N, D538G) (4, 53). These residues and their phosphorylation are essential in controlling the agonist state of the LBD domain of ER, conformational changes, and protein stability (54, 55), thus mutations at these residues lead to altering of the conformational dynamics of the loop connecting Helix 11 and Helix 12 in the LBD of ER, which leads to a stabilized agonist state, even though there is endocrine treatment (56). The Y537 and D538 ER mutants are also phosphorylated on S118 by the TFIIH kinase, cyclin-dependent kinase (CDK)7 in an estrogen-independent manner, which may be the possible reason for endocrine resistance by potentiating transcriptional activity of mutant ER-driven cancer (55). Mutations in the ER gene also confer loss of ER function, which is also associated with endocrine resistance (3). Targeting the transcriptional function of mutant ER proteins using BET inhibitor OTX015 is effective in reversing endocrine therapy resistance due to ER mutations (57). This indicates drugs that reverse ER mutation could be the possible treatment strategy in endocrine-resistant breast cancer resulting from ER mutation.

#### Altered Expression Patterns of Co-Regulatory Proteins

The transcriptional role of ER depends on co-regulatory proteins, which may activate (coactivators) or inhibit (corepressors) ER-driven transcription (3). The role of coactivators and corepressors in endocrine resistance is discussed below.

##### Coactivators

The SRC/p160 family of nuclear receptor coactivators are the best-characterized coactivators and consist of three members. These are SRC-1/NCoA-1, SRC-2/GRIP1/TIF2/NCoA-2, and SRC-3 (p/CIP, RAC3, ACTR, AIB1, and TRAM-1) (1, 58). These coactivators have several functional domains, such as NH2-terminal basic helix-loop-helix-Per/Ah receptor nuclear translocation/Sim domain, receptor-interacting domain (RID), and carboxyl-terminal activation domains 1 (AD1) and 2 (AD2). The RID is responsible for binding with ER and activates ER targeted gene transcription. The AD1 is capable of interacting with histone acetyltransferase (HAT) CBP and p300. The CBP and p300 are also involved in chromatin remodeling and able to activate ligand-induced ER function (59). The AD 2 can interact with protein arginine methyltransferases (PRMT), such as coactivator-associated arginine methyltransferase-1 (CARM-1) and PRMT-1 (59), which relax chromatin structure and increase the accessibility of basal components of the transcriptional machinery to ER target genes (60).

The SRC-1 initiates the transcription of endocrine-resistant genes independent of the ER (61). Coactivators like SRC-1 coordinate several signaling pathways, and that makes it an important player in tumor cells to escape endocrine therapy (62). SRC also promotes tamoxifen resistance by up-regulating SIRT1 (63).

The SRC-3 mRNA overexpression is also associated with tamoxifen resistance (37, 58, 64). Knockdown of SRC-3 can restore the antitumor effects of tamoxifen (65). The overexpression of AIB1-Δ3 isoform may contribute to antiestrogen resistance (66). The expression of SRC3 is also promoted by the general control non-derepressible 5 (GCN5), which leads to tamoxifen resistance by reducing p53 levels (67). Coactivators like SRC to play a significant role in the different signaling pathways, therefore using SRC inhibitors like small molecule inhibitors (SMIs) in combination with other treatments will be the best treatment strategy in endocrine-resistant ER-positive breast cancer.

##### Corepressors

Several transcriptional corepressors are involved in breast cancer, but nuclear corepressors (NCOR1), NCOR2, and the nuclear receptor subfamily 2, group F, member 2 (NR2F2) are the best-characterized corepressors (68).

##### Nuclear Receptor Corepressor 1

Nuclear receptor corepressor 1, also known as retinoid X receptor-interacting protein-13 (RIP-13) is a 270 kDa protein and has three RIDs found on the c-terminal region of NCOR1. The RIDs are responsible for the direct interactions between ERs and the repression domains (RI, RII, and RIII) found in the N-terminus region, which are responsible for the repressive functions. The binding of estrogen to ERs induces the movement of the H12 that stabilizes NCOR1 interactions (59, 69).

The downregulation of NCOR1 has been associated with tamoxifen resistance (6, 37, 70). Reduced levels of NCOR1 relieve the inhibition of MYC, CCND1, and SDF1 gene transcription and result in tamoxifen behaving as a partial agonist for cell cycle progression. It also regulates chromatin accessibility by recruiting histone deacetylase (HDAC)3, which leads to histone deacetylation, chromatin condensation, and loss of RNA polymerase II that induces repression of basal gene transcription (68, 71).

##### Nuclear Receptor Corepressor 2

Nuclear receptor corepressor 2 is also known as the silencing mediator for retinoid or thyroid hormone receptors (SMRTs) shows 41% amino acid sequence similarity with NCoR1 (68). It has four known repression domains within the N-terminal portion (RD1, RD2, RD3, and RD4) and two C-terminal nuclear receptor interaction domains (RID1 and RID2) (72). The RIDs are responsible for direct binding with ER and form a dimer on its 290-427 and 1788-1903 amino acids region and recruits other co-repressors such as GPS2, TBLR1, HDAC3, which suppress the pro-proliferative ER signaling pathway (72). Similar to NCOR1, the low-level expression of NCOR2 is related to tamoxifen resistance (68).

##### Nuclear Receptor Subfamily 2, Group F, Member 2

The NR2F2 gene that encodes chicken ovalbumin upstream promoter transcription factor 2 (COUP-TFII) displays copy number loss in 21% of ER-positive breast cancer. The direct interaction of COUP-TFII with dimerized ER prevents ER-dependent gene transcription and also allows it to recruit other corepressors, such as NCOR1 and NCOR2 that enhance repression. The antiproliferative effects of tamoxifen are enhanced by COUP-TFII overexpression (68). Similar to other corepressors, the expression of NR2F2 is also decreased in tamoxifen resistance (73).

#### Alteration of Transcriptional Factors

Transcriptional factors such as SP-1, AP-1, and NFκB are important for indirect/non-classical pathways to regulate the transcription of genes that do not contain EREs (20, 24). An increased expression of these transcriptional factors is associated with tamoxifen resistance in ER-positive breast cancer (74).

#### Role of MicroRNAs

MicroRNAs (miRNAs) are small non-coding RNA molecules that regulate the expression of genes by degrading mRNA or suppressing translation (6). The oncogenic and tumor suppressor miRNAs are important for inducing and inhibiting cancer cell proliferation and invasion, respectively (75). Loss and modification of ER by miRNAs is the main mechanism of endocrine therapy resistance (75). They also inhibit the translation of the target mRNA (76). The miR-21 promotes tamoxifen resistance by targeting PTEN. Tamoxifen resistance due to miR-221/222 has also resulted *via* p27 and ER modulation, thereby enabling tumor growth in an ER-independent manner. The miR-155 also promotes tamoxifen resistance *via* suppression of cytokine signaling 6 (77). Other miRNAs that induce tamoxifen resistance include miR-181b, miR-101, miR-301and miR-519a, while miR-342, miR-116, miR-10a, miR-15a/16, miR-200b, miR-200c, miR-375, miR-261, miR-575, and miR-451 are also responsible for suppressing tamoxifen resistance (78). MiR-148a and miR-152 have also reduced tamoxifen resistance in ER breast cancer *via* downregulating ALCAM (79).

#### Role of Extracellular Vehicles

Extracellular vehicles are secreted particles that carry DNA, RNA, and protein, and are capable of transferring information and activities onto receptive cells. Extracellular vehicle treatment activates AP-1 and NF-κB transcription factors which were implicated in hormone therapy resistance in breast cancer. Exosome-mediated resistance is also achieved by the activation of the phosphoinositide 3-kinase (PI3K)/AKT pathway (6). Plasma circulating exosomes derived from obese women could also lead to tamoxifen resistance (80).

Extracellular vehicles also transport P-glycoprotein, which expels drugs located in the cytoplasm to the extracellular surface, and are the main mechanism of endocrine resistance. Transport of miR-221/222 by extracellular vehicles is also responsible for endocrine resistance (81, 82). Exporting larger quantities of drugs into the extracellular surface of the cell is seen in drug-resistant cells than drug-sensitive cells (83). The exosome mediated transfer of urothelial carcinoma-associated 1 (UCA1) also significantly increases tamoxifen resistance (84). Treatment of the parent MCF-7 cells with exosomes from the resistant cells also leads to the partial resistance of the MCF-7 cells to antiestrogen drugs (85).

#### Receptor Tyrosine Kinases

##### Human Epidermal Growth Factor 2 Signaling

Signaling through human epidermal growth factor 2 (HER2) influences the genomic actions mediated by ER. Increased crosstalk between ER and HER2 coupled with high expression of coactivator steroid receptor coactivator-3 (SRC3) is suggested as one of the endocrine drug resistance mechanisms (3). HER2, through its transcriptional regulator PEA3, contributes to endocrine resistance by potentiating steroid coactivator proteins (86).

Tamoxifen behaves as an estrogen agonist in breast cancer cells that express high levels of HER2 (87). Overexpression of HER2 and its downstream MAPK may contribute to the loss of ER, which is directly attributed to endocrine resistance (3). HER-2 overexpression also increases the anti-apoptotic Bcl-2 and BclxL proteins, which results in the reduction of tamoxifen-induced apoptosis and boosts tamoxifen resistance (44). The silencing of SRC3 or inhibiting the activity of HER2 can resensitize cells to tamoxifen treatment (3). Ubiquitin ligase c-Cbl can reverse tamoxifen resistance in HER2-overexpressing breast cancer cells by inhibiting the formation of the ER-SRC-HER2 complex (88).

##### Insulin-Like Growth Factor-1 Receptor

Insulin-like growth factor-1 receptor (IGF-1R) is indicated in breast cancer development, progression, and metastasis through its involvement in Ras/Raf/MEK1/2/ERK1/2 and PI3K/AKT/mTOR pathway (89). The interaction of this receptor with ER results in the redistribution of ER from the nucleus to extranuclear areas and increases ligand-independent activation of ER, which further activates Ras/Raf/MEK1/2/ERK1/2 and PI3K/AKT/mTOR pathway and results in acquired endocrine resistance (90).

##### Fibroblast Growth Factor Receptor

Similar to other RTK, fibroblast growth factor receptor (FGFR) families have also been implicated in breast cancer development and progression. High expression of fibroblast growth factor receptor 3 (FGFR3) is indicated in tamoxifen-resistant breast tumors by stimulating activation of the Ras/Raf/MEK1/2/ERK1/2 and PI3K/AKT/mTOR signaling pathways (91). Amplification of fibroblast growth factor receptor 1(FGFR1) also promotes cyclin D1 expression in ER-positive breast cancer, resulting in resistance to antiestrogen (92). Fibroblast growth factor receptor 2 (FGFR2) was also identified as a mediator of FGF7 action and associated with resistance to tamoxifen (93).

#### Cell Cycle Regulators

##### Over-Expression of Positive Regulators

The c-Myc is a well-known cell cycle regulator and oncogene frequently up-regulated in breast cancer (94). Over-expression of the positive regulators such as c-Myc and cyclins E1 and D1 are involved in endocrine resistance by activating cyclin-dependent kinases (37). The c-Myc expression is required for the estrogen-independent proliferation of breast cancer cells expressing ERαY537S and ERαD538G mutations and the c-Myc alone is sufficient to confer antiestrogen resistance in human breast cancer (95). The role of c-Myc in endocrine resistance may be linked with HSPC111 (HBV pre-S2 trans-regulated protein 3) (96, 97). The combinatorial use of cell cycle inhibitors along with hormonal therapy represents could be a novel therapeutic modality (94).

##### Reduced Expression of Negative Regulators

Reduced expression of a negative regulator such as p21 and p27 is associated with tamoxifen resistance (37). A recent study implicated loss of p21 function as one possible cause of tamoxifen-resistant (98). The loss of p21 was associated with a tamoxifen growth-inducing phenotype (99). Similar to p21, p27 inhibition has recently been associated with a tamoxifen-resistance (100). The absence of p21 enabled cyclin-CDK complexes to aberrantly phosphorylate ER when bound to tamoxifen, resulting in a growth-stimulatory phenotype (101).

### Metabolic Resistance

The cytochrome P2D6 (CYP2D6) enzyme metabolizes a quarter of all prescribed drugs and is one of the main enzymes responsible for converting tamoxifen into its active metabolites (102). Endocrine therapy specifically tamoxifen is a prodrug that requires metabolism to form the pharmacologically active metabolites such as N-desmethyltamoxifen and 4-hydroxytamoxifen by cytochrome P450 (CYP)-mediated catalysis (3). Cytochrome P3A4/5 and 2D6 are major CYP isozymes involved in tamoxifen metabolism. Polymorphism of CYP2D6 also affects the metabolism of tamoxifen and leads to tamoxifen resistance (103).

Retrospective clinical data suggests that specific single nucleotide polymorphisms (SNPs) of CYP2D6 can lead to null or reduced enzyme activity resulting in worse outcomes for those individuals when treated with tamoxifen. Selective serotonin reuptake inhibitor antidepressant drugs such as paroxetine and fluoxetine have potently inhibited the metabolism of tamoxifen by CYP2D6 and thus potentially may lessen the efficacy of tamoxifen (104).

Increasing the efflux of the cell that leads to a decrease in intracellular concentrations of a drug is a general mechanism of drug resistance. Reduced uptake of tamoxifen from extracellular sources and the lower availability of intracellular tamoxifen could confer resistance (3). Extracts from resistant tumors had on average a 10-fold lower tamoxifen concentration than sensitive tumors (105). Although the precise mechanisms for lower intracellular tamoxifen levels remain unclear, potential mechanisms include the presence of microsomal antiestrogen binding sites (AEBSs) which bind tamoxifen with a similar high affinity as the ER and increase tamoxifen efflux *via* multi-drug resistance (MDR) P-glycoprotein drug pump (3, 106).

### Immune System-Dependent Resistance

Increasing the level of immune cell-like tumor-associated macrophage and soluble mediators like interleukin-1B (IL-1B) and tumor necrosis factor α (TNFα) are important for predicting poor prognosis in breast cancer. The tumor necrosis factor α (TNFα) and Il-1B activates the nuclear factor kappa B (NF-κB) leads to endocrine resistance (4). The activity of NF‐κB is suppressed by COUP-TFII and decreased COUP-TFII expression results in an endocrine-resistant phenotype (107). The NF‐κB signaling pathway inhibitor (ACT001) in combination with tamoxifen hinders the proliferation of tamoxifen-resistant cells (108), hence NF-κB inhibition plays a promising approach to prevent tamoxifen resistance (109).

The IL-1b induces epithelial-mesenchymal transition by activation of the IL-1b/IL-1RI/b-catenin pathway, the up-regulation of TWIST1 leads to methylation of the estrogen receptor 1 gene promoter. This epigenetic modification produced a significant decrease of the ER receptor levels and increased resistance to tamoxifen (110). The expression of quiescent ALDH+ IL1R1+ cancer stem cell population that activates the IL1β signaling pathway is increasing following antiestrogen therapy and acts as an adaptive strategy that facilitates treatment resistance (111).

The chemokine (C-C motif) ligand 2 (CCL2) secreted by tumor-associated macrophages (TAM) activates PI3K/Akt/mTOR signaling and promotes an endocrine resistance feedback loop in the tumor microenvironment (TME), suggesting that CCL2 and TAM may be novel therapeutic targets for patients with endocrine-resistant breast cancer (112). The chemokine (C-C motif) ligand 2 (CCL2) secreted by tumor-associated macrophages activates PI3K/Akt/mTOR signaling and promotes an endocrine resistance feedback loop in the tumor microenvironment (TME), suggesting that CCL2 and tumor-associated macrophages may be novel therapeutic targets for patients with endocrine-resistant breast cancer (112). Transforming growth factor-beta (TGFβ) also contributes to the development of antiestrogen resistance (113).

Macrophage infiltration results in increased TNF and IL1B signaling, which stimulates aromatase expression, resulting in increased estrogen levels and increased ER signaling in ER-positive breast cancer and associated with tamoxifen resistance (114, 115).

## Conclusions

The mechanisms of endocrine resistance to ER-positive breast cancer remain a mystery. Even though it is still at the early stage, several mechanisms of endocrine resistance are identified in the above review, which are basic and crucial insights for developing drugs that target interconnected pathways. Our review indicates that a combination of endocrine therapy with other drugs that target different molecular pathways and coregulators will be one of the most promising treatment approaches.

## Author Contributions

All authors contributed to the article and approved the submitted version.

## Funding

This study was funded by Mizan Tepi University, Addis Ababa University, and the Armauer Hansen Research Institute (AHRI).

## Conflict of Interest

The authors declare that the research was conducted in the absence of any commercial or financial relationships that could be construed as a potential conflict of interest.

## Abbreviations

AD, Activation domains; AF1, Activation Function 1; AF-2, Activation Function 2; AIB1, Amplified in Breast Cancer 1; AP-1, Activator Protein; BLACAT1, Bladder Cancer-associated Transcript 1; CARM-1, Coactivator-associated arginine methyltransferase-1; CBP, CREB Binding Protein ; CCL2, Chemokine (C-C motif) Ligand 2; CDK, Cyclin-dependent kinase; CYP2D6, Cytochrome P450/2D6; DBD, DNA Binding Domain; ER, Estrogen receptor; EREs, Estrogen Response Elements; ERα, Estrogen Receptor α; ERβ, Estrogen Receptor β; FGFR, Fibroblast Growth Factor Receptor; GCN5, General control non-derepressible 5; GPR30, G protein-coupled receptor 30; H12, Helix 12; HAT, Histone acetyltransferase; HDAC, Histone deacetylase; HSP27, Heat shock protein 27; IGF, Insulin-like growth factor; IGF-1R, Insulin-like growth factor-1 receptor; IL-1B, Interleukin-1B; LBD, Ligand-binding Domain; MAPK, Mitogen-activated Protein Kinase; CoA, Coactivator; CoR, Corepressor; NCOR, Nuclear corepressors; NR2F2, Nuclear receptor subfamily 2, group F, member 2; PI3K, Phosphatidylinositol 3 kinases; PKA, Protein Kinase A; PRMT, Protein arginine methyltransferase; PTEN, Phosphatase and tensin homolog deleted on chromosome ten; RIDs, Receptor interaction domain; RTK, Receptor tyrosine kinase; SERDs, Selective ER Down-regulators; SERMs, Selective ER Modulators; SMRT, Silencing Mediator of Retinoic Acid and Thyroid Hormone Receptor; Sp1, Specificity Protein 1; TAM, tumor-associated macrophages; TGFβ, Transforming Growth Factor-beta; TME, Tumor microenvironment; TNFα, Tumor Necrosis Factor α; TSC, Tuberous sclerosis.
