# Corrigendum: Molecular Mechanisms of Endocrine Resistance in Estrogen-Receptor-Positive Breast Cancer

**DOI:** 10.3389/fendo.2021.689705

**Published:** 2021-05-11

**Authors:** Esmael Besufikad Belachew, Dareskedar Tsehay Sewasew

**Affiliations:** ^1^ Biology, Mizan Tepi University, Addis Ababa, Ethiopia; ^2^ Microbial, Cellular and Molecular Biology Department, Addis Ababa University, Addis Ababa, Ethiopia; ^3^ Immunology, Armauer Hansen Research Institute (AHRI), Addis Ababa, Ethiopia

**Keywords:** acquired resistance, breast cancer, endocrine resistance, endocrine therapy, estrogen receptor, *de novo* resistance

In the original article, there was an erroring in the title, which was missing the word “Receptor”.

A correction has been made to the title as follows: Molecular Mechanisms of Endocrine Resistance in Estrogen-Receptor-Positive Breast Cancer.

The authors apologize for this error and state that this does not change the scientific conclusions of the article in any way. The original article has been updated.

